# Clade distribution of *Candida auris* in South Africa using whole genome sequencing of clinical and environmental isolates

**DOI:** 10.1080/22221751.2021.1944323

**Published:** 2021-07-01

**Authors:** Serisha D. Naicker, Tsidiso G. Maphanga, Nancy A. Chow, Mushal Allam, Stanford Kwenda, Arshad Ismail, Nelesh P. Govender

**Affiliations:** a National Institute for Communicable Diseases (Centre for Healthcare-Associated Infections, Antimicrobial Resistance and Mycoses), a Division of the National Health Laboratory Service, Johannesburg, South Africa; b School of Pathology, Faculty of Health Sciences, University of the Witwatersrand, Johannesburg, South Africa; c Mycotic Diseases Branch, Centers for Disease Control and Prevention, Atlanta, GA, USA; d National Institute for Communicable Diseases (Core Sequencing Facility), a Division of the National Health Laboratory Service, Johannesburg, South Africa; e Division of Medical Microbiology, Faculty of Health Sciences, University of Cape Town, Cape Town, South Africa

**Keywords:** *Candida auris*, whole genome sequencing, clades, South Africa, single nucleotide polymorphisms

## Abstract

In South Africa, *Candida auris* was the third most common cause of candidemia in 2016–2017. We performed single nucleotide polymorphism (SNP) genome-wide analysis of 115 *C. auris* isolates collected between 2009 and 2018 from national laboratory-based surveillance, an environmental survey at four hospitals and a colonization study during a neonatal unit outbreak. The first known South African *C. auris* strain from 2009 clustered in clade IV. Overall, 98 strains clustered within clade III (85%), 14 within clade I (12%) and three within clade IV (3%). All environmental and colonizing strains clustered in clade III. We also identified known clade-specific resistance mutations in the *ERG11* and *FKS1* genes. Identification of clade I strains between 2016 and 2018 suggests introductions from South Asia followed by local transmission. SNP analysis characterized most *C. auris* strains into clade III, the clade first reported from South Africa, but the presence of clades I and IV strains also suggest early introductions from other regions.

## Introduction

*Candida auris* has emerged as an important human pathogen in the last decade [1]. This yeast is difficult to identify using standard microbiological techniques [2], is almost universally resistant to fluconazole and variably susceptible to the echinocandins and amphotericin B [3], and causes outbreaks in healthcare settings [4].

*C. auris* was first described in 2009, although the earliest known case of infection was retrospectively identified from an archived blood culture isolate collected in 1996 [5]. A recent discovery of *C. auris* in a remote coastal wetland suggests that this fungus may have existed in the natural environment before its emergence and recognition as a human pathogen [6]. Over the last decade, there has been an exponential increase in the number of reported cases globally and *C. auris* has now been identified in more than 40 countries on six continents [1]. In South Africa, *C. auris* was the third most common cause of candidemia in 2016–2017 [7]. *C. auris* is most closely related to *Candida lusitaniae* and *Candida haemulonii* based on DNA sequencing of the 28S ribosomal gene [1]. Whole genome sequencing (WGS)-based phylogenetic analysis divides *C. auris* into five populations, namely clade I (South Asian), clade II (East Asian), clade III (African), clade IV (South American) and clade V (Iranian) [3,8].

Each clade has varying characteristics such as aggregate formation, pseudohyphae production, growth on media containing cycloheximide, antifungal susceptibility [9] and antifungal resistance gene mutations [3]. Initial WGS analysis of *C. auris* strains collected between 2012 and 2015 from Pakistan, India, Venezuela and South Africa showed clustering of strains by geographic region suggesting that each clade emerged independently. In this early analysis, the 10 South African isolates were separated by fewer than 70 single nucleotide polymorphisms (SNPs) between any two isolates but there were thousands of SNPs between clades [3]. These South African strains were the first identified clade III strains. WGS analysis has also identified clade-specific point mutations in the *FKS1* and *ERG11* genes that have been linked to resistance to echinocandins and fluconazole respectively [3,10]. In a more recent analysis of 304 isolates from 24 countries, much more phylogeographic mixing of highly-related isolates within clades was observed across countries [10]. This suggested multiple introductions into some countries, probably related to cross-border travel of people with prior exposure to *C. auris* in a healthcare setting. Based on a Bayesian clock phylogeny, outbreak-causing fluconazole-resistant clade III isolates emerged approximately 37 years ago [10]. Thus identification of clade III in southern Africa decades later does not necessarily mean that the clade originated in this region.

We performed WGS SNP analysis of 115 South African *C. auris* genomes collected from national laboratory-based surveillance, an environmental survey conducted at four hospitals and a colonization study during a neonatal unit outbreak to describe the clade distribution and molecular epidemiology of *C. auris* in South Africa. We also identified known clade-specific resistance mutations in the *ERG11* and *FKS1* genes.

## Materials and methods

### Isolate selection

#### National laboratory-based surveillance for candidemia

The National Institute for Communicable Diseases (NICD) conducted national laboratory-based surveillance for candidemia from February 2009 through August 2010 [11]. We included the first South African *C. auris* strain which was retrospectively identified through this surveillance [12]. National laboratory-based surveillance for candidemia was repeated from January 2016 through December 2017 and 400 viable *C. auris* isolates were submitted to a reference laboratory for identification and antifungal susceptibility testing, as previously described [7]. We randomly selected a nationally-representative sample of 92 of 400 isolates for WGS from this 2016–2017 survey. This number included five patients with two or more serial isolates.

#### Environmental survey

We performed an environmental survey during 2017 in the intensive care units of four private-sector hospitals in Gauteng Province, South Africa, two of which had reported outbreaks of *C. auris* infection and two with no known outbreaks. We used a systematic sampling approach whereby we swabbed “high-touch” and other defined surfaces in patient care areas. Ten *C. auris* strains were identified from the two hospitals with known outbreaks. We isolated *C. auris* from the hands of a healthcare worker, a handwashing basin, bed linen and bed rails, a windowsill, a curtain, a drying rack and on the floor around a bed. *C. auris* isolates were identified by matrix-assisted laser desorption/ionisation time-of-flight (MALDI-TOF) mass spectrometry (Bruker, Germany) and we included all 10 environmental strains in our study.

#### Colonization survey

Following detection of an outbreak of *C. auris* bloodstream infections in the neonatal unit of a public-sector hospital in Gauteng Province [13], we determined the *C. auris* colonization status of 31 admitted babies on a single day in September 2017. We identified 11 *C. auris* strains from axilla/groin swabs by standard microbiology techniques and MALDI-TOF mass spectrometry and included these in our study.

#### National laboratory-based surveillance for Candida auris

National laboratory-based surveillance for *C. auris* infections was initiated on 1 August 2018 in South Africa and all public- and private-sector pathology laboratories were requested to submit any confirmed or suspected *C. auris* isolated from any specimen site. We only included one *C. auris* strain isolated in 2018 from urine from this surveillance since this isolate had high minimum inhibitory concentration (MIC) values for fluconazole (256 µg/ml), anidulafungin and micafungin (≥4 µg/ml) and amphotericin B (4 µg/ml) and was considered pan-resistant.

### Species identification and antifungal susceptibility testing

Reference laboratory identification and susceptibility testing methods were the same as described previously [7,14–16]. Briefly, MALDI-TOF mass spectrometry was performed to confirm species-level identification [7]. For antifungal susceptibility testing (AFST), the MICs of nine antifungal agents (amphotericin B, fluconazole, voriconazole, itraconazole, posaconazole, caspofungin, anidulafungin, micafungin and flucytosine) were determined using commercial dried broth microdilution panels containing Alamar blue (Thermo Fisher Scientific, Cleveland, OH, USA) according to Clinical and Laboratory Standards Institute M27-A3 and M60 recommendations [14–16]. Additionally, amphotericin B MICs were determined by Etest (bioMérieux, Marcy l’Etoile, France) on RPMI 1640 plates containing 2% glucose (Diagnostic Media Products [DMP], NHLS, Johannesburg, South Africa). We performed AFST on clinical surveillance isolates only. Tentative US CDC breakpoints were used to interpret MICs [17].

### Processing of isolates for WGS

Isolates were retrieved from −70°C freezer storage and sub-cultured onto Sabouraud agar (DMP) to confirm viability. Thereafter isolates were sub-cultured onto chromogenic agar (DMP) to confirm purity. DNA was extracted using the ZR Fungal/Bacterial DNA Miniprep kit (Zymo Research, Irvine, California). Before isolates were sent for WGS at the NICD Core Sequencing Facility (NSCF), sequencing of the *ITS* region was performed to confirm that all isolates were *C. auris*.

### WGS SNP and resistance gene mutation analysis

Paired-end libraries were prepared using the Nextera DNA Flex library preparation kit, followed by 2 × 300 bp sequencing on a MiSeq instrument (Illumina, San Diego, CA, USA). FastQC and PRINSEQ were used to assess read data quality and perform read filtering [18]. Read data were then aligned using Burrows–Wheeler Aligner (BWA) to a South African *C. auris* strain (B11221, NCBI BioSample number: SAMN05379609) previously sequenced on the PacBio platform by Lockhart et al., in 2017 [3]. We also included genomes of seven South African strains that had been sequenced previously [3]. We also added external genomes for each of the clades. For clade I: PEKT02 (B8441, NCBI BioSample number: SAMN05379624) and SRR3883445 (B11214, NCBI BioSample number: SAMN05379602). For clade II: PYFR01 (B11220, NCBI BioSample number: SAMN05379608). For clade IV: PYGM01 (B11243, NCBI BioSample number: SAMN05379619). For clade V: SRR9007776 (NCBI BioSample number: SAMN11570381). SNP variants were identified using SAMtools. BWA and SAMtools were performed using the publicly available SNP analysis pipeline NASP [19]; filtering parameters involved removing positions that had <10x coverage, <90% variant allele calls and those within duplicated regions in the reference [18]. Phylogenetic analysis was performed using MEGA whereby a maximum parsimony tree with 500 bootstrap replicates was constructed. A targeted gene approach using CLC Genomics Workbench version 10 (Qiagen, The Netherlands) was performed on surveillance isolates to determine known resistance gene mutations in *ERG11* and *FKS1* [10]. All WGS data are in the NCBI SRA under BioProject accession number PRJNA737309.

### Epidemic curve analysis

We plotted an epidemic curve of cases of *C. auris* bloodstream infection by clade (I, III, IV and not sequenced) and collection date (2016–2017). We also plotted an epidemic curve by clade (I, III, IV and not sequenced) and facility for the same time period.

### Ethics

For national laboratory-based surveillance and outbreak investigation activities, annual ethics approvals were sought and obtained from several university ethics committees in South Africa.

## Results

**Figure 1.**
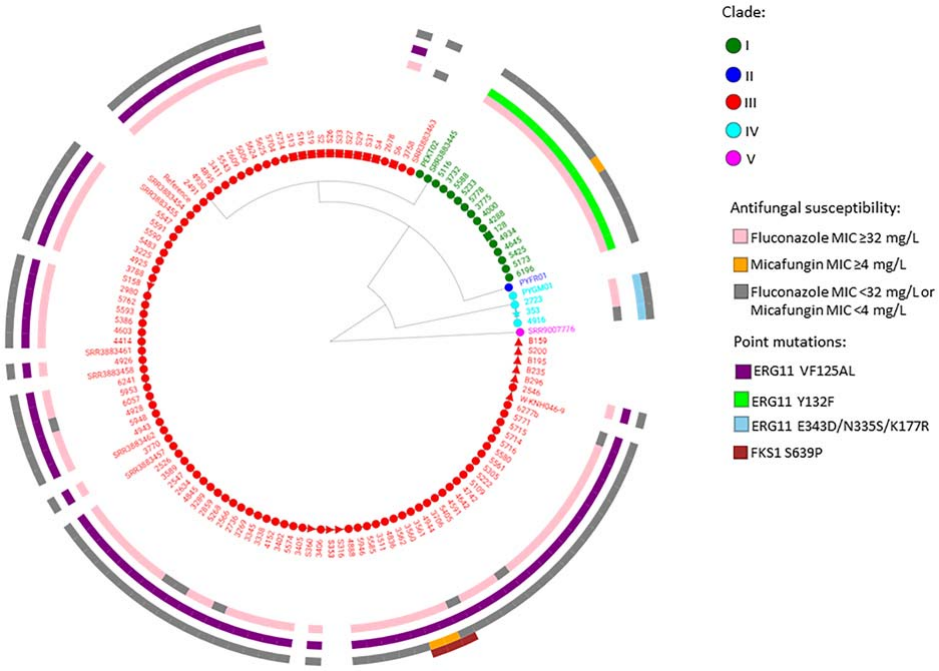
WGS SNP and resistance gene mutation analysis of 115 invasive, environmental and colonizing South African *Candida auris* strains collected between 2009 and 2018 during national laboratory-based candidemia and *Candida auris* surveillance, an environmental survey and a neonatal unit outbreak. There were 211 626 SNPs in total with a coverage breadth of 62% and this is an unrooted maximum parsimony tree with 500 bootstrap replicates. The consistency index was 0.89 and the number of phylogenetic sites were 211 626. Circles represent invasive isolates, red squares represent colonizing strains and the green square represents the one isolate from urine in 2018. The red triangles represent the environmental strains and the light blue star represents the first *C. auris* strain isolated in 2009.

Ninety-eight South African *C. auris* strains clustered within clade III (85%, African clade), 14 *C. auris* strains were in clade I (12%, South Asian clade) and three strains were in clade IV (3%, South American clade) as shown in Figure 1. The first known invasive strain isolated in 2009 from a patient admitted to a public-sector hospital in Gauteng Province clustered in clade IV. The 92 invasive strains, isolated from patients admitted to both public- and private-sector hospitals in 2016-2017, clustered in clades III (77; 84%), I (13; 14%) and IV (2; 2%). All 10 strains from the environmental survey conducted at four private-sector hospitals in 2017 clustered in clade III. All 11 strains from the colonization survey at a neonatal unit in 2017 also clustered in clade III. The pan-resistant *C. auris* strain isolated in 2018 from urine of a patient admitted to a private-sector hospital clustered in clade I. There were 105 sequenced *C. auris* isolates collected from 98 patients in our study. Characteristics of these 98 patients are shown in Table 1. Most patients were adults admitted to private-sector hospitals in Gauteng Province.

**Table 1.** Characteristics of 98 South African patients infected or colonized with *Candida auris* strains sequenced in this study.

Characteristic	Number (%)*
Sex	
Male	55 (63)
Female	32 (37)
Age, years (median [IQR])	52 (38–62)
<1	15 (16)
1–17	2 (2)
18–44	29 (30)
45–64	34 (35)
≥65	16 (17)
Year of diagnosis	
2009	1 (1)
2016	32 (33)
2017	64 (65)
2018	1 (1)
Specimen type	** **
Blood	86 (88)
Axilla and groin swabs (skin colonization)	11 (11)
Urine	1 (1)
Health sector	
Private	68 (69)
Public	30 (31)
Province	
Gauteng	93 (95)
KwaZulu-Natal	2 (2)
Free State	1 (1)
Limpopo	1 (1)
Mpumalanga	1 (1)

Note: IQR: interquartile range.

*Missing data for sex and age.

**Figure 2.**
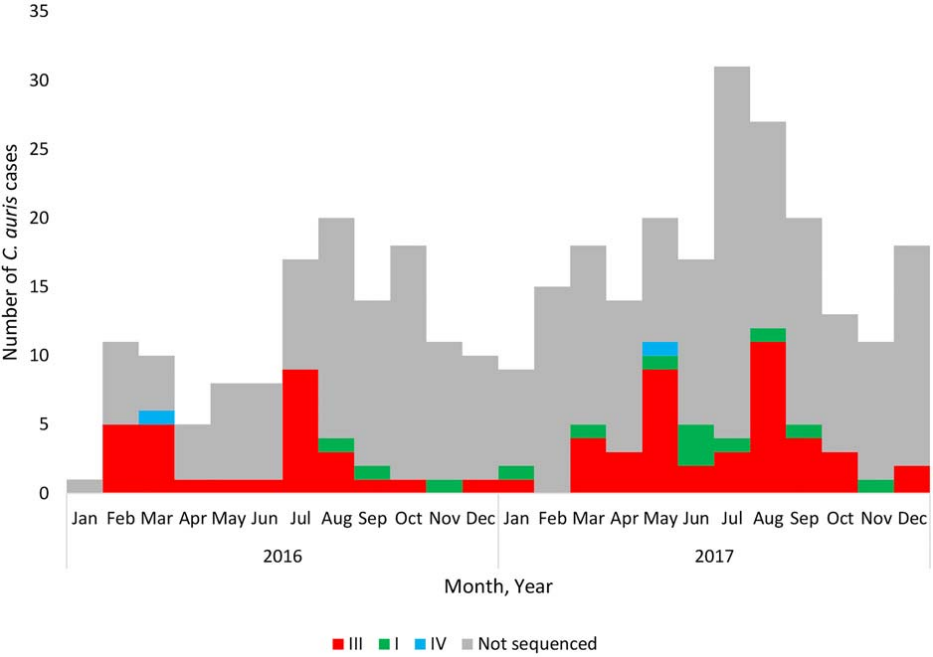
Epidemic curve of cases of *Candida auris* bloodstream infection by clade (I – green, III – red, IV – blue and not sequenced – grey) and collection date (2016–2017).

**Figure 3.**
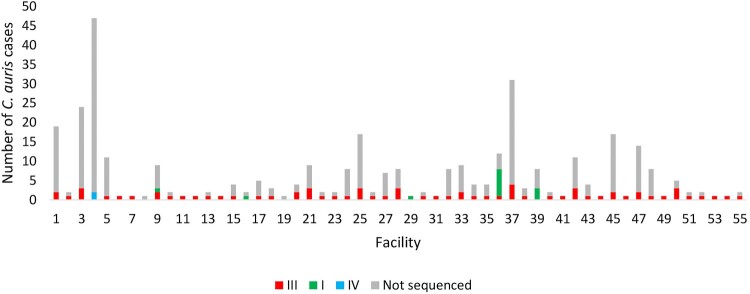
Epidemic curve of cases of *Candida auris* bloodstream infection by clade (I – green, III – red, IV – blue and not sequenced – grey) and acute-care hospital facility (collection dates: 2016–2017).

 Figure 2 shows an epidemic curve of all clinical cases of bloodstream *C. auris* infection detected by national surveillance in 2016 and 2017 plotted by clade and collection date. There were ≥20 sequenced/non-sequenced *C. auris* cases identified in August 2016, May 2017 and between July-September 2017, indicating peaks of an ongoing outbreak during these months in 2016–2017. Figure 3 shows the epidemic curve of clinical cases of bloodstream infection detected by national surveillance in 2016 and 2017 plotted by clade (I, III, IV and not sequenced) and facility. There were two facilities in Gauteng Province (one public-sector [Facility 9] and one private-sector [Facility 36]) where both clade I and III isolates were identified from patients admitted to these hospitals (Figure 3). All sequenced isolates from four hospitals (Facilities 4, 16, 29 and 39) belonged to a clade other than clade III (either clade I or clade IV).

The first known occurrence of a clade III isolate was in October 2012 [3]. In clade III, there were 77 invasive isolates obtained from 70 patients during national candidemia surveillance (2016–2017). The characteristics of these 70 patients are shown in Supplementary Table 1. Most patients were adults admitted to private-sector hospitals in the Gauteng province. The minimum, median and maximum number of SNPs within clade III were 0, 23 and 71 respectively. There were no SNP differences between the serial isolates collected from five patients. The MIC distribution of the 77 invasive isolates in clade III collected during national surveillance in 2016–2017 are shown in Table 2. Seventy isolates (91%) were resistant to at least one antifungal agent and 69 (90%) clade III isolates had a fluconazole MIC of ≥32 µg/ml. Seventy six (99%) clade III isolates also had the VF125AL mutation in the *ERG11* gene (Figure 1 and Table 4). Of the 69 fluconazole-resistant isolates, seven (9%) also had an amphotericin B MIC of ≥2 µg/ml and were therefore multidrug resistant. Two out of three clade III isolates collected from the same patient were considered pan-resistant; both strains had micafungin MICs of ≥4 µg/ml, amphotericin B MICs of ≥2 µg/ml and fluconazole MICs of ≥32 µg/ml. All three strains had the S639P mutation in the hot spot 1 region of the *FKS1* gene (Figure 1 and Table 4).

**Table 2.** Antifungal susceptibility distribution of *Candida auris* strains within clade III collected during the 2016–2017 national laboratory-based surveillance for candidemia, *n *= 77.

Antifungal agent	Test method	Number of isolates with MIC (mg/L) of:																
		0.015	0.03	0.06	0.125	0.25	0.5	1	2	4	8	16	32	64	128	256	MIC_50_	MIC_90_
Itraconazole	BMD		2	9	42	20	3	1									0.12	0.25
Voriconazole	BMD		1	2	3	5	19	36	9	1	1						1	2
Posaconazole	BMD	5	19	32	15	4	2										0.06	0.12
Fluconazole	BMD									1	2	5	11	10	24	24	128	256
Caspofungin	BMD		5	43	22	5					1	1					0.06	0.12
Micafungin	BMD		2	36	34	1	1		1	1	1						0.12	0.12
Anidulafungin	BMD		2	18	40	14		2	1								0.12	0.25
Flucytosine	BMD			16	42	17	1	1									0.12	0.25
Amphotericin B	BMD						5	49	23								1	2
Amphotericin B	Etest	3	2	3	4	19	26	10	4	5	1						0.38	2

Notes: BMD: broth microdilution, MIC: minimum inhibitory concentration (mg/L).

All environmental and colonizing strains clustered in clade III. We compared the number of SNPs between environmental strains and invasive strains collected from the two private-sector hospitals (Facilities 15 and 20). There were fewer than 27 SNPs between any of the six environmental strains and one invasive strain collected from Facility 15. There were fewer than 15 SNPs between any of the four environmental strains and any of the four invasive strains collected from Facility 20. We also compared the number of SNPs between colonizing strains and invasive strains collected from one public-sector hospital (Facility 50). There were fewer than five SNPs between any of the four invasive strains that were sequenced from neonatal cases of *C. auris* candidemia and any of the 11 colonizing strains collected during the neonatal unit outbreak at Facility 50.

The first known appearance of a clade I isolate was in August 2016. The 14 strains in clade I were collected between 2016 and 2018. Thirteen South African clade I strains were from patients admitted to private-sector hospitals in Gauteng Province and one patient was admitted to a public-sector hospital in the same province. Eight patients were male and six patients were female. The minimum, median and maximum number of SNPs for clade I South African strains (n=14) were 0, 11 and 28 respectively. There were fewer than 50 SNPs between any South African isolate and the Indian strain accession number SRR3883445 and more than 600 SNPs between any South African strain and the Pakistan strain PEKT02. The ranges, MIC_50_ and MIC_90_ values for all antifungals are shown in Table 3. All 14 strains had high MIC values to fluconazole (≥128 µg/ml) with the Y132F mutation in the *ERG11* gene (Figure 1 and Table 4). Twelve of the 14 strains (86%) also had an amphotericin B MIC of ≥2 µg/ml and were therefore multidrug resistant. The pan-resistant strain from 2018 also belonged to clade I.

**Table 3.** Antifungal susceptibility distribution of *Candida auris* strains within clades I (*n* = 14) and IV (*n* = 3) from this study.

Antifungal agent	Clade I			Clade IV		
	Range	MIC_50_	MIC_90_	Range	MIC_50_	MIC_90_
Itraconazole	0.12–0.25	0.25	0.25	0.06–0.5	0.12	0.5
Voriconazole	1–2	2	2	0.12–0.5	0.25	0.5
Posaconazole	0.03–0.25	0.12	0.12	0.03–0.25	0.25	0.25
Fluconazole	128–256	256	256	16 – 64	64	64
Caspofungin	0.12–16	0.5	2	0.12–0.5	0.5	0.5
Micafungin	0.12–8	0.12	0.5	0.12–0.25	0.25	0.25
Anidulafungin	0.12–4	0.25	1	0.12–0.25	0.25	0.25
Flucytosine	0.06–0.25	0.12	0.12	0.06–0.12	0.12	0.12
Amphotericin B						
By BMD	2–4	2	4	1–2	1	2
By Etest	0.5–4	2	3	0.5–0.75	0.75	0.75

Notes: BMD: broth microdilution, MIC: minimum inhibitory concentration (mg/L).

**Table 4.** Proportion of fluconazole-resistant and micafungin-resistant *Candida auris* strains with resistance gene mutations by clade from this study.

Clade	Fluconazole resistance				Micafungin resistance	
	MIC ≥32 mg/L (n/N, %)	*ERG11* mutations			MIC ≥4 mg/L (n/N, %)	*FKS1HP1* mutation
		VF125AL	Y132F	E343D/ N335S/ K177A		S639P
I	14/14 (100%)	–	14/14 (100%)	–	1/14 (7%)	**–**
III	69/77 (90%)	76/77 (99%)	–	–	2/77 (3%)	3/3 (100%)
IV	2/3 (67%)	–	–	3/3 (100%)	**–**	**–**

Note: MIC – minimum inhibitory concentration (mg/L).

The three clade IV strains were identified from a single public-sector hospital in Gauteng; one isolate was isolated in November 2009, one in March 2016 and one in May 2017 (Figure 2). Strains in clade IV were isolated from two adult male patients and one pediatric male patient who were all admitted to the burns unit at a single public-sector hospital (Facility 4 in Figure 3). There were fewer than 20 SNPs among any South African isolate in clade IV but fewer than 250 SNPs between any South African strain and the Venezuelan strain PYGM01. The ranges, MIC_50_ and MIC_90_ values for all antifungals are shown in Table 3. All three isolates in clade IV had high fluconazole MIC values (range: 16–64 µg/ml) but were susceptible to echinocandins (range: 0.12–0.25 µg/ml) and amphotericin B (range: 0.5–0.75 µg/ml). All three clade IV strains had three mutations (E343D, N335S and K177A) in the *ERG11* gene (Figure 1 and Table 4).

## Discussion

The first known *C. auris* strain from South Africa, which was isolated in 2009, clustered in clade IV suggesting an early introduction of *C. auris* from South America although the reverse could also be possible. We found evidence that clade IV isolates spread in one South African public-sector hospital (Facility 4) since two strains isolated seven to eight years later from the same hospital also clustered in clade IV with <20 SNPs. Clade IV, however, did not appear to spread widely across South African hospitals since 84% of our nationally-representative sample of bloodstream *C. auris* strains from 2016 to 2017 clustered in clade III. We found 14 clade I strains between 2016 and 2018 suggesting one or more introductions from South Asia followed by local transmission (14% of isolates in the 2016–2017 survey). We found that most South African *C. auris* strains were highly related and clustered in clade III (African).

We found co-circulation of three clades (clades I, III and IV) in South Africa during a 9-year period (2009–2018). With international travel, this has been observed in other countries such as Canada (clades I, II and III), Kenya (clades I and III) and United States (clades I, II, III and IV) [10]. Based on reports in the literature, clade I is the most widely distributed and has been found in Canada, France, Germany, India, Kenya, Pakistan, Saudi Arabia, the United Kingdom, the United Arab Emirates and the United States. Clade II has been reported from Canada, Japan, South Korea and the United States. Clade III has been reported from Australia, Canada, Kenya, South Africa, Spain, the United Kingdom and the United States. Clade IV is found in Colombia, Israel, Panama, the United States and Venezuela. Clade V isolates have only been found in Iran so far [10]. We found co-circulation of clade I and clade III isolates at three hospitals. Two of these three hospitals are in close proximity (about three kilometres apart) and patients may have been transferred between hospitals. The travel histories of patients in our study as well as whether patients received health care abroad was not known making it difficult to determine how introductions of clades I and IV occurred. It is also possible that healthcare workers colonized with clade I or IV strains moved between facilities and then transmitted these strains to patients.

We randomly selected *C. auris* strains from candidemia surveillance during 2016–2017 to generate a representative sample. Most isolates were from patients admitted to private-sector hospitals in the Gauteng Province. This distribution was also observed in a larger national study of candidaemia during 2016–2017 [7]. Indiscriminate use of azoles and echinocandins, and inadequate infection prevention and control measures may have led to the emergence and subsequent establishment of *C. auris* in acute-care hospitals in this province [7,20,21]. Clade III was the dominant clade in our study. This clade seems to have spread throughout South Africa since strains within this clade were identified from patients admitted to approximately 50 hospitals across the country. The colonizing and environmental strains from hospitals with active outbreaks were also found to be within this clade. Chow and colleagues observed that fewer than 12 SNPs suggest recent transmission of *C. auris* [22]. We found fewer than five SNPs between the invasive and colonizing *C. auris* strains in the neonatal unit outbreak indicating recent clonal transmission. Certain phenotypic characteristics of clade III strains, for example aggregate formation, may allow for persistence in the environment [9]. However, it is not known whether these phenotypic differences may enable clade III strains to dominate transmission and hence cause outbreaks [9].

Clade I isolates were more resistant to fluconazole and micafungin than clade III and IV isolates in our study, which has been observed previously [23]. Clade-specific mutations were observed within the *ERG11* and *FKS1* genes for each of the clades identified in our study. Our clade III isolates also had the S639 mutation in the hot spot 1 region of the *FKS1* gene, this mutation has only been previously observed in isolates from clades I and IV [10,24].

Since we studied a larger number of strains, this is the first report of South African strains belonging to clades I and IV but these clades probably had limited transmission after introduction into South Africa. Clade I isolates were identified from patients admitted to hospitals in Gauteng Province suggesting that some local transmission has happened. Interestingly, the South African *C. auris* strain identified in 2009 clustered in clade IV suggesting a possible South American origin although the travel history of the patient was unknown. We only have evidence of clade IV isolates circulating in one South African hospital (Facility 4); this hospital has also experienced a recent outbreak of *C. auris* infections in its neonatal department. There were only two strains randomly selected from this particular hospital and a further analysis of a larger number of *C. auris* strains from this hospital is currently being performed to determine if all strains cluster in clade IV. There were no clade II isolates found in our study. However, clade II isolates are the least prevalent globally [25] and our sample mostly consisted of bloodstream *C. auris* strains rather than isolates from other sampling sites. A larger WGS study of strains from an ongoing national laboratory-based surveillance for *C. auris* infections (all specimen sites) would allow us to determine the prevalence of clade II isolates in South Africa.

Our large study genetically characterized the majority of South African *C. auris* strains into clade III but there were a small number of strains in clades I and IV. We were able to trace the emergence of *C. auris* in South Africa and this study provides a baseline for future WGS studies of larger numbers of *C. auris* strains.

## Supplementary Material

Clean_copy_of_supplementary_material.docx

## Members of the TRAC-South Africa group (2009–2010)

NHLS Chris Hani Baragwanath: Jeannette Wadula; van Rensburg and partners: Chris Janse van Rensburg; NHLS Groote Schuur: Andrew Whitelaw; Ampath National Laboratory Service: Inge Zietsman (co-principal investigator), Norman Miller, Peter Smith, Johan van Greune, Adrian Brink; NHLS Steve Biko Pretoria Academic: Anwar Hoosen, NHLS Charlotte Maxeke Johannesburg Academic: Olga Perovic; NHLS Dr George Mukhari: Maphoshane Nchabaleng; NHLS Tygerberg: Heidi Orth; NHLS Inkosi Albert Luthuli: Yacoob Coovadia; NHLS Universitas: Loekie Badenhorst; Lancet laboratories: Johan Moolman, AK Peer, Chetna Govind; NHLS Helen Joseph/Rahima Moosa: Ranmini Kularatne, Barry Bhagoobhai; Vermaak and partners: Ben Prinsloo; NHLS Grey’s: Sumayya Haffejee; NHLS Greenpoint: John Simpson; NHLS Rob Ferreira: Greta Hoyland; PathCare laboratories: Marthinus van Schalkwyk; NHLS East London: Glenda Bowie; NHLS Mthatha: Patricia Hanise, Sandeep Vasaikar; NHLS Pelonomi: Linda Wende; US Centers for Disease Control and Prevention: Tom Chiller, Angela Ahlquist-Cleveland, Shawn Lockhart; National Institute for Communicable Diseases: Jaymati Patel, Nelesh P. Govender (principal investigator).

## Members of GERMS-SA (2012–2020)

John Black, Vanessa Pearce (Eastern Cape); Masego Moncho, Motlatji Maloba (Free State); Caroline Maluleka, Charl Verwey, Charles Feldman, Colin Menezes, David Moore, Gary Reubenson, Jeannette Wadula, Merika Tsitsi, Maphoshane Nchabeleng, Nicolette du Plessis, Nontombi Mbelle, Nontuthuko Maningi, Prudence Ive, Theunis Avenant, Trusha Nana, Vindana Chibabhai (Gauteng); Adhil Maharj, Fathima Naby, Halima Dawood, Khine Swe Swe Han, Koleka Mlisana, Lisha Sookan, Nomonde Mvelase, Praksha Ramjathan, Prasha Mahabeer, Romola Naidoo, Sumayya Haffejee, Surendra Sirkar (Kwazulu Natal); Ken Hamese, Ngoaka Sibiya, Ruth Lekalakala (Limpopo); Greta Hoyland, Sindi Ntuli (Mpumalanga); Pieter Jooste (Northern Cape); Ebrahim Variava, Ignatius Khantsi (North West); Adrian Brink, Elizabeth Prentice, Kessendri Reddy, Andrew Whitelaw (Western Cape); Ebrahim Hoosien, Inge Zietsman, Terry Marshall, Xoliswa Poswa (AMPATH); Chetna Govind, Juanita Smit, Keshree Pillay, Sharona Seetharam, Victoria Howell (LANCET); Catherine Samuel, Marthinus Senekal (PathCare); Andries Dreyer, Khatija Ahmed, Louis Marcus, Warren Lowman (Vermaak and Vennote); Anne von Gottberg, Anthony Smith, Azwifarwi Mathunjwa, Cecilia Miller, Charlotte Sriruttan, Cheryl Cohen, Desiree du Plessis, Erika van Schalkwyk, Farzana Ismail, Frans Radebe, Gillian Hunt, Husna Ismail, Jacqueline Weyer, Jackie Kleynhans, Jenny Rossouw, John Frean, Joy Ebonwu, Judith Mwansa-Kambafwile, Juno Thomas, Kerrigan McCarthy, Liliwe Shuping, Linda de Gouveia, Linda Erasmus, Lynn Morris, Lucille Blumberg, Marshagne Smith, Martha Makgoba, Mignon du Plessis, Mimmy Ngomane, Myra Moremi, Nazir Ismail, Nelesh Govender, Neo Legare, Nicola Page, Nombulelo Hoho, Ntsieni Ramalwa, Olga Perovic, Portia Mutevedzi, Ranmini Kularatne, Rudzani Mathebula, Ruth Mpembe, Sibongile Walaza, Sunnieboy Njikho, Susan Meiring, Tiisetso Lebaka, Vanessa Quan, Wendy Ngubane (NICD).
