# Supplementary material for: Clade distribution of Candida auris in South Africa using whole genome sequencing of clinical and environmental isolates
Source: Emerg Microbes Infect. 2021 Jul 1;10(1):1300–8. doi: 10.1080/22221751.2021.1944323 (PMC8253216; doi:10.1080/22221751.2021.1944323)
Supplement: Clean_copy_of_supplementary_material.docx [file TEMI_A_1944323_SM1212.docx]

**Supplementary Table 1:** Characteristics of 70 South African patients infected with *Candida auris* clade III strains collected during national laboratory-based candidemia surveillance, 2016-2017

| **Characteristic** | **Number (%)*** |
| --- | --- |
| **Sex** |  |
| Male | 44 (63) |
| Female | 26 (37) |
| **Age, years (median [IQR])** | 52 (37-62) |
| <1 | 4 (6) |
| 1-17 | 2 (3) |
| 18-44 | 23 (34) |
| 45-64 | 27 (40) |
| ≥65 | 12 (18) |
| **Health sector** |  |
| Private | 55 (79) |
| Public | 15 (21) |
| **Ward type** |  |
| Adult | 64 (91) |
| Paediatric | 4 (6) |
| Unknown | 2 (3) |
| **Province** |  |
| Gauteng | 65 (93) |
| KwaZulu-Natal | 2 (3) |
| Free State | 1 (1.5) |
| Limpopo | 1 (1.5) |
| Mpumalanga | 1 (1.5) |

IQR: interquartile range; *Missing data for age
